# NF-κB is activated in response to temozolomide in an AKT-dependent manner and confers protection against the growth suppressive effect of the drug

**DOI:** 10.1186/1479-5876-10-252

**Published:** 2012-12-21

**Authors:** Simona Caporali, Lauretta Levati, Grazia Graziani, Alessia Muzi, Maria Grazia Atzori, Enzo Bonmassar, Giuseppe Palmieri, Paolo A Ascierto, Stefania D’Atri

**Affiliations:** 1 Laboratory of Molecular Oncology, Istituto Dermopatico dell’Immacolata-IRCCS, Via dei Monti di Creta 104, Rome, 00167, Italy; 2 Department of System Medicine, University of Rome “Tor Vergata”, Via Montpellier 1, Rome, 00133, Italy; 3 Institute of Translational Pharmacology, National Research Council, Via Fosso del Cavaliere 100, Rome, 00133, Italy; 4 Unit of Cancer Genetics, Institute of Biomolecular Chemistry, National Research Council, Traversa La Crucca 3, Baldinca Li Punti, Sassari, 07100, Italy; 5 Department of Melanoma, Unit of Melanoma, Cancer Immunotherapy and Innovative Therapies, Istituto Nazionale Tumori Fondazione “G. Pascale”, Via Mariano Semmola, Naples, 80131, Italy

**Keywords:** Nuclear factor κB, AKT, Temozolomide, NEMO binding domain peptide, Cell proliferation, Cell senescence

## Abstract

**Background:**

Most DNA-damaging chemotherapeutic agents activate the transcription factor nuclear factor κB (NF-κB). However, NF-κB activation can either protect from or contribute to the growth suppressive effects of the agent. We previously showed that the DNA-methylating drug temozolomide (TMZ) activates AKT, a positive modulator of NF-κB, in a mismatch repair (MMR) system-dependent manner. Here we investigated whether NF-κB is activated by TMZ and whether AKT is involved in this molecular event. We also evaluated the functional consequence of inhibiting NF-κB on tumor cell response to TMZ.

**Methods:**

AKT phosphorylation, NF-κB transcriptional activity, IκB-α degradation, NF-κB2/p52 generation, and RelA and NF-κB2/p52 nuclear translocation were investigated in TMZ-treated MMR-deficient (HCT116, 293TLα^-^) and/or MMR-proficient (HCT116/3-6, 293TLα^+^, M10) cells. AKT involvement in TMZ-induced activation of NF-κB was addressed in HCT116/3-6 and M10 cells transiently transfected with AKT1-targeting siRNA or using the isogenic MMR-proficient cell lines pUSE2 and KD12, expressing wild type or kinase-dead mutant AKT1. The effects of inhibiting NF-κB on sensitivity to TMZ were investigated in HCT116/3-6 and M10 cells using the NF-κB inhibitor NEMO-binding domain (NBD) peptide or an anti-RelA siRNA.

**Results:**

TMZ enhanced NF-κB transcriptional activity, activated AKT, induced IκB-α degradation and RelA nuclear translocation in HCT116/3-6 and M10 but not in HCT116 cells. In M10 cells, TMZ promoted NF-κB2/p52 generation and nuclear translocation and enhanced the secretion of IL-8 and MCP-1. TMZ induced RelA nuclear translocation also in 293TLα^+^ but not in 293TLα^-^ cells. AKT1 silencing inhibited TMZ-induced IκB-α degradation and NF-κB2/p52 generation. Up-regulation of NF-κB transcriptional activity and nuclear translocation of RelA and NF-κB2/p52 in response to TMZ were impaired in KD12 cells. RelA silencing in HCT116/3-6 and M10 cells increased TMZ-induced growth suppression. In M10 cells NBD peptide reduced basal NF-κB activity, abrogated TMZ-induced up-regulation of NF-κB activity and increased sensitivity to TMZ. In HCT116/3-6 cells, the combined treatment with NBD peptide and TMZ produced additive growth inhibitory effects.

**Conclusion:**

NF-κB is activated in response to TMZ in a MMR- and AKT-dependent manner and confers protection against drug-induced cell growth inhibition. Our findings suggest that a clinical benefit could be obtained by combining TMZ with NF-κB inhibitors.

## Background

The nuclear factor κB (NF-κB) family of transcription factors regulates the expression of multiple target genes involved in a variety of physiological and pathological processes, including inflammation, innate and adaptive immune response, angiogenesis, tumorigenesis, and metastasis [1-3].

In mammals, the NF-κB family is composed of RelA (p65), c-Rel, RelB, NF-κB1 (p50) and NF-κB2 (p52), which can form homo- and heterodimers. The latter two family members are produced as precursor proteins, p105 and p100, respectively, which are subjected to cotranslational (p105) or stimulus-dependent (p100) processing to the mature forms [1-3].

In resting cells, NF-κB homo- and heterodimers are maintained latent in the cytoplasm through the association with inhibitory proteins (inhibitors of κB, IκBs). In response to a variety of signals which act through membrane and/or cytoplasmic receptors (e.g. pro-inflammatory cytokines, ligands for Toll-like receptors, engagement of B and T cell receptors), IκBs are degraded rendering NF-κB free to translocate into the nucleus and regulate gene transcription [1-3]. In this regard, two main pathways of NF-κB activation can be recognized, namely the “canonical” and the “non-canonical” pathway, both converging on the activation of the IκB kinase (IKK) complex. The IKK complex, in turn, phosphorylates IκBs marking them for ubiquitin-dependent degradation [1,2,4]. NF-κB activation through the canonical pathway involves the IKK complex comprising the catalytic subunits IKK-α and IKK-β as well as the regulatory subunit NF-κB essential modulator (NEMO/IKKγ) and, in most cells, leads to IκB-α degradation and nuclear translocation of p50/RelA dimers [1-3]. The non-canonical pathway, predominantly active in B cells, is typically triggered by some members of the TNF cytokine family and it has been suggested to be dependent on the activation of IKK-α homodimers [1,2,4]. In this pathway, which does not require the function of NEMO, activation of IKK leads to phosphorylation and proteolytic cleavage of p100 to produce the mature NF-κB2/p52 subunit. Subsequently, p52/RelB dimers translocate into the nucleus [1,2,4].

The canonical and non-canonical pathways of NF-κB activation can also be engaged by a number of genotoxic agents [5-8]. The signal transduction mechanisms linking DNA damage in the nucleus with NF-κB activation in the cytoplasm have not been fully elucidated yet, but they appear to require the function of ATM (Ataxia-Telangiectasia Mutated) and NEMO [5-8]. Notably, NF-κB activation in response to DNA damage can result in either protection from or sensitization to the genotoxic agent, depending on the cell line and the nature and/or amount of the agent [5-8].

Temozolomide (TMZ) is a methylating triazene compound approved for the treatment of newly diagnosed glioblastoma and refractory anaplastic astrocytoma [9,10]. Although not approved for this indication, TMZ is frequently used off-label for the treatment of metastatic melanoma. Currently, a number of clinical trials are evaluating TMZ in combination therapies for other solid tumors, including colorectal cancer (http://www.clinicaltrials.gov).

The antitumor activity of TMZ is primarily due to the methylation of the O^6^ position of guanine (O^6^-G) in DNA. If not repaired, O^6^-methylguanine (O^6^-MeG) frequently mispairs with thymine during DNA duplication. O^6^-MeG:T mismatches then trigger the intervention of the mismatch repair (MMR) system [11,12], which, however, fails to find a correct partner for O^6^-MeG. As a consequence, futile cycles of repair generates nicks in the DNA and activates a signaling cascade resulting in cell cycle arrest at the G_2_ phase of the second cell doubling event, which is followed by either apoptosis, mitotic catastrophe, or a senescence-like state [12-19]. Methyl adducts at O^6^-G are specifically removed by the DNA repair protein O^6^-methylguanine-DNA methyltransferase (MGMT) [20,21]. Therefore, MMR-proficient cells endowed with elevated MGMT activity are resistant to TMZ, but they can be sensitized to the drug by MGMT inhibitors [e.g. O^6^-benzylguanine (BG)] [20,21]. MMR-deficient cells are, instead, highly resistant to TMZ regardless of their MGMT activity (Additional file 1: Figure S1).

The serine/threonine kinase AKT is a critical regulator of major cellular processes, including gene expression, glycogen metabolism, migration, proliferation, and survival [22]. Once activated, AKT controls cellular functions through phosphorylation of a large variety of substrates, including several molecules involved in the NF-κB signaling pathways [1,3,23,24]. Recently, we have demonstrated that in colon and breast cancer cells, treatment with TMZ provokes AKT activation that counteracts the growth suppressive effects of the drug [25]. Therefore, in the present study we investigated whether TMZ-induced activation of AKT results in the triggering of the NF-κB signalling pathway. Moreover, we evaluated whether NF-κB inhibition, achieved through either RNA interference technology or the use of a selective NF-κB inhibitor, is able to increase tumor cell sensitivity to TMZ.

## Methods

### Cell lines

The MMR-proficient human metastatic melanoma cell line M10 [26] was kindly provided by Dr. D. Del Bufalo (Regina Elena Cancer Institute, Rome, Italy) and cultured in GIBCO™ RPMI-1640 medium (Invitrogen Corporation, Carlsbad, CA) supplemented with 10% fetal calf serum (FCS) (GIBCO®), 2 mM GIBCO™ L-Glutamine, and 50 μg/ml GIBCO™ Gentamicin.

The MMR-deficient human colorectal cancer cell line HCT116 and its MMR-proficient subline HCT116/3-6 were kindly provided by Dr. G. Marra (Institute of Molecular Cancer Research, University of Zurich, Zurich, Switzerland). HCT116 cells have a hemizygous nonsense mutation in the *hMLH1* gene located on chromosome 3 [27]. The HCT116/3-6 subline was created by microcell chromosome transfer of a single normal human chromosome 3 into HCT116 cells [28]. The cell lines were maintained in McCoy’s 5A medium (Sigma-Aldrich, St. Louis, MO) supplemented with 10% FCS (GIBCO®), 2 mM GIBCO™ L-Glutamine, 50 μg/ml GIBCO™ Gentamicin and, in the case of HCT116/3-6 cells, 400 μg/ml G418 (Sigma-Aldrich).

The human embryonic kidney (HEK) 293T-MutLα^-^/Lα^+^ cell line (hereafter referred to as 293T±Lα) was a gift of Prof. J. Jiricny (Institute of Molecular Cancer Research, University of Zurich, Zurich, Switzerland). The cell line was derived from the hMLH1-deficient cells HEK293T [29] by stable transfection with a vector carrying the *hMLH1* cDNA under the control of the inducible Tet-Off expression system [29]. In the absence of doxycycline the cell line expresses the hMLH1 protein. Conversely, in the presence of the drug the transcription of *hMLH1* is turned off [29]. The cell line was grown in DMEM with Eagle salts (GIBCO®), supplemented with 10% Tet-System approved FCS (BD Biosciences Pharmigen, San Diego, CA), 2 mM GIBCO™ L-Glutamine, 50 μg/ml GIBCO™ Gentamicin, 100 μg/ml Zeocin (Invitrogen Corporation), and 300 μg/ml Hygromycin B (Roche Applied Science, Mannheim, Germany). To turn off hMLH1 expression the cells were cultured for 7 days in the presence of 50 ng/ml doxycycline (Sigma-Aldrich).

The MCF-7/KD12 cell line (hereafter referred to as KD12) was generated by stable transfection of an expression vector encoding a dominant-negative kinase-dead form of AKT1 (mutation K179M) into the MCF-7/B1 clone, derived from the MMR-proficient breast cancer cell line MCF-7 [25]. The MCF-7/pUSE2 cell line (hereafter referred to as pUSE2) was obtained by transfection of the empty vector into the same cell clone. Both cell lines were maintained in the culture medium described for M10 cells, supplemented with 400 μg/ml G418.

M10, HCT116, HCT116/3-6, and MCF-7 cell lines were previously shown to be MGMT-proficient [30-32]. The 293T±Lα cell line does not express MGMT [29].

HCT116 and MCF-7 cells have been previously screened for mutation in *HRAS*, *NRAS*, *KRAS*, *BRAF* and *PIK3CA*[33]. HCT116 cells were found to harbor a heterozygous oncogenic mutation in *KRAS* (p.G13D) and *PIK3CA* (p.H1047R), whereas MCF-7 cells were found to be mutated in *PIK3CA* (p.E545K) [33]. To assess whether constitutive activation of the mitogen-activated protein kinase (MAPK) and/or PI3K/AKT signalling pathways was also present in M10 cells, we performed a mutational analysis of *NRAS*, *BRAF* and *PIK3CA* in these cells. Briefly, the complete coding regions and intron-exon boundaries of exons 2 and 3 in *NRAS*, exon 15 in *BRAF*, and exons 9 and 20 in *PIK3CA* were directly sequenced using an automated fluorescence-based cycle sequencer (ABI PRISM® 3100, Life Technologies, Carlsbad, CA) as previously described [34]. The M10 cell line was found to be mutated in *BRAF* (p.V600E) and wild-type in *NRAS* and *PIK3CA*.

### Drugs and reagents

TMZ was kindly provided by Merck Sharp & Dohme-Merck & Co., Inc. (Whitehouse Station, NJ) and was always prepared freshly in culture medium, because the drug readily decomposes in aqueous solution. The NF-κB-inhibitor NEMO-binding domain (NBD) peptide [35] was purchased from Alexis Corporation (San Diego, CA) as a solution of 10 mM in phosphate buffered saline (PBS). The MGMT-inhibitor BG was purchased from Sigma-Aldrich and dissolved in ethanol (2.4 mg/ml). Both inhibitors were stored as stock solutions at −80°C, and diluted in culture medium just before use. In all assays, with the exception of those performed with 293T±Lα cells, BG was used at the final concentration of 10 μM, added to the cells 2 h before TMZ and left into the culture up to the end of TMZ treatment. At the concentration used, BG did not affect cell proliferation (data not shown). 3-(4,5-dimethylthiazol-2-yl)-2,5-diphenyltetrazolium bromide (MTT), and 4’-6-Diamidino-2-phenylindole (DAPI) were purchased from Sigma-Aldrich. MTT was prepared at a concentration of 5 mg/ml in PBS, and stored at 4°C.

Reagents for SDS-polyacrylamide gel electrophoresis were all purchased from Bio-Rad Laboratories (Hercules, CA).

### NF-κB luciferase reporter assay

HCT116/3-6, HCT116, M10, pUSE2 and KD12 cells were seeded into 24-well plates (BD Falcon™, Becton, Dickinson and Company, Franklin Lakes, NJ) and allowed to adhere at 37°C for 18 h. The cells were then transiently co-transfected with 3 μg (M10) or 0.075 μg (HCT116/3-6, HCT116) or 0.3 μg (pUSE2 and KD12) of the NFκB-responsive firefly luciferase reporter pNF-κB-Luc (Stratagene, La Jolla, CA) and 250 ng (M10) or 6.25 ng (HCT116/3-6, HCT116) or 25 ng (pUSE2 and KD12) of the *Renilla* luciferase expression vector pRL-null (Promega Corporation, Madison, WI) using Lipofectamine™ 2000 Reagent (Invitrogen Corporation) according to the manufacturer’s protocol. Twenty-four hours after transfection, cells were exposed to 50 μM TMZ plus BG or to BG alone as a control. After 48 and 72 h of culture, the cells were lysed and luciferase assays were performed using the Dual-Luciferase® Reporter Assay (Promega) according to the manufacturer’s instructions. For each sample, firefly luciferase activity was normalized to *Renilla* luciferase activity and then to the total protein amount used in the assay.

To evaluate the effect of NBD peptide on NF-κB activity, M10 cells were plated and co-transfected with the pNF-kB-Luc and pRL-null vectors as described above. Twenty-four hours after transfection, 50 μM NBD peptide was added to the cultures. After additional 24 h of incubation, untreated and NBD peptide-treated cells were exposed to 50 μM TMZ plus BG or to BG alone. Seventy-two hours after drug addition, the cells were lysed and luciferase assays were performed as described above.

### Enzyme-Linked Immunosorbent Assay (ELISA)

M10 cells were seeded in 100-mm plates (BD Falcon™) and allowed to adhere at 37°C for 18 h. The cells were then exposed to 50 μM TMZ plus BG or BG alone for 48 or 72 h. The amounts of interleukin 8 (IL-8) and monocyte chemoattractant protein-1 (MCP-1) released into the culture medium were determined by ELISA using the OptEIA Set for human IL-8 or MCP-1 (BD Biosciences Pharmigen) according to the manufacturer's instructions. Proteins were normalized to the number of total cells counted in each culture at the time of supernatant collection.

### Western blot analysis

Rabbit polyclonal antibodies against AKT and phospho-AKT (Ser473) were purchased from Cell Signaling Technology, Inc. (Beverly, MA). Rabbit polyclonal antibodies against IκB-α (C-21), NF-κB2/p52 (K27) and RelA (C20), as well as mouse monoclonal antibody (mAb) against lamin A/C (346) were purchased from Santa Cruz Biotechnology, Inc., (Santa Cruz, CA). mAb against actin (Clone AC-40) was obtained from Sigma-Aldrich.

Whole cell extracts and nuclear extracts were prepared as described previously [18,36]. Twenty-five μg (whole cell extracts) or 10 μg (nuclear extracts) of proteins per sample were run on a 10% SDS-polyacrilamide gels, transferred to nitrocellulose membranes (Amersham Biosciences, Buckinghamshire, UK) and blocked with 5% non-fat milk in Tris-buffered saline supplemented with 0.1% Tween 20 for 1 h at room temperature. The membranes were then incubated in the same solution overnight at 4°C with primary antibodies at the following dilutions: anti-AKT 1:1000; anti-phospho-AKT 1:500; anti-IκB-α 1:200; anti-NF-κB2/p52 1:200; anti-RelA 1:500; anti-lamin A/C 1:500, anti-actin 1:1000. The latter two antibodies were used as internal standards for loading. Immunodetection was carried out using appropriate horseradish peroxidase-linked secondary antibodies and enhanced chemiluminescence (ECL) detection reagents (Amersham Biosciences). Where indicated, films were scanned on a GS-710 Calibrated Imaging Densitometer and analyzed by means of Quantity One Software Version 4.1.1 (Bio-Rad Laboratories).

### Transient transfection with small interfering RNA (siRNA) targeting RelA

Oligonucleotide siRNA targeting RelA (AGCACAGAUACCACCAAGA) (siNFp65) and the corresponding scramble oligonucleotide (GAUCGCGAGCCAAACUAUA) (scrNFp65) to be used as a control were purchased from Sigma-Proligo (The Woodlands, TX).

For Western blot analysis, HCT116/3-6 and M10 cells were suspended in culture medium without antibiotics, seeded into 60-mm dishes (BD Falcon™) and allowed to adhere at 37°C for 18 h. The cells were then transfected with 100 nM siNFp65 or scrNFp65 using Oligofectamine™ Reagent (Invitrogen Corporation) according to the manufacturer’s protocol. As an additional control group, the cells were treated with the transfection reagent alone (mock-transfected cells). After 72 h of culture at 37°C, the cells were recovered, plated and subjected to a second transfection as described above. Total cell extracts were prepared 7 days after the second transfection.

For chemosensitivity assays, HCT116/3-6 and M10 cells recovered after the first transfection were seeded into 96-well plates (BD Falcon™), allowed to adhere at 37°C for 18 h, and then subjected to the second transfection. After 24 h of incubation, the cells were exposed to graded concentrations of TMZ plus BG or to BG alone. The plates were incubated at 37°C for 6 days and cell proliferation was then evaluated by the MTT assay, as previously described [31]. Four replica wells were used for each group. TMZ concentration producing 50% inhibition of cell growth (i.e. IC_50_), was calculated on the regression line in which absorbance values at 595 nm were plotted against the logarithm of drug concentration.

### Transient transfection with siRNA targeting AKT1

Oligonucleotide siRNA targeting AKT1 (CUCACAGCCCUGAAGUACU) (siAKT1) and the corresponding scramble oligonucleotide (GAUCCUAUAUUCGGUUAGU) (scrAKT1) to be used as a control were purchased from Sigma-Proligo.

For Western blot analysis, M10 and HCT116/3-6 cells were suspended in culture medium without antibiotics, seeded into 6-well plates (BD Falcon™), allowed to adhere at 37°C for 18 h, and then transfected with 50 nM siAKT1 or scrAKT1 using Lipofectamine™ RNAiMAX Reagent. (Invitrogen Corporation). Twenty-four hours after transfection, the cells were incubated with 50 μM TMZ plus BG or with BG alone. Whole cell extracts were prepared after 72 h of TMZ exposure.

### Treatment with NBD peptide for proliferation assays

To evaluate the effect of NBD peptide on cell proliferation, M10 and HCT116/3-6 cells were seeded into 96-well plates (BD Falcon™), allowed to adhere at 37°C for 18 h, and then incubated with concentrations of NBD peptide ranging between 12.5 and 100 μM. After 6 days of culture, cell proliferation was evaluated by the MTT assay, and the IC_50_ values of NBD peptide were determined. The mean IC_50_ value of NBD peptide was 63.93 μM (four independent experiments, standard error of the mean: 3.16 μM) and 72.97 μM (three independent experiments, standard error of the mean: 4.03 μM) for M10 and HCT116/3-6 cells, respectively. On this basis, the NBD concentration of 50 μM, producing about 30-35% of growth inhibition in both cell lines, was selected for the subsequent studies.

To investigate the effects of NBD peptide on cell sensitivity to TMZ, M10 and HCT116/3-6 cells were plated and allowed to adhere as described above, and then left untreated or exposed to 50 μM NBD peptide for 24 h. Thereafter, the cells were incubated with graded concentrations of TMZ plus BG or with BG alone. Cell proliferation was evaluated by the MTT assay after 5 days of TMZ exposure.

### Senescence-associated β-galactosidase (SA-β-Gal) staining

M10 and HCT116/3-6 cells were plated into 24-well plates (BD Falcon™), allowed to adhere at 37°C for 18 h and then left untreated or exposed to 50 μM NBD peptide for 24 h. The cells were then incubated with 50 μM TMZ plus BG or with BG alone for 4 days. At the end of the incubation period, the cells were fixed and stained using the Senescence β-Galactosidase Staining Kit from Cell Signaling Technology Inc., according to the manufacturer’s protocol. The percentage of SA-β-Gal positive cells was determined by counting five different randomly selected fields (each comprising 50–150 cells) per samples under a bright-field microscope (10x magnification).

### Analysis of senescence-associated heterochromatin foci (SAHF)

M10 and HCT116/3-6 cells were grown on 8-well Lab-Tek® II chamber slides (ThermoFisher Scientific, Rochester, NY) for 18 h and then left untreated or exposed to 50 μM NBD peptide for 24 h. Afterward, the cells were incubated with 50 μM TMZ plus BG or with BG alone for 7 days, and then fixed with 4% paraformaldehyde. After washing with PBS, the cells were permeabilized with 0.2% Triton-X-100/PBS at room temperature for 10 min. The cells were then washed twice with PBS, incubated with 1 μg/ml DAPI at room temperature for 1 min, and washed again in PBS. Slides were mounted in a 90% glycerol/PBS solution and examined using a fluorescence microscope (Axioskop 2 microscope and AxioCam camera, Zeiss, Thornwood, NY).

### Statistical analysis

Statistical significance among different TMZ IC_50_ values, different levels of IL-8 and MCP-1 secretion, and different percentage of senescent cells was assessed using Student’s *t* test analysis. Statistical significance among different values of firefly luciferase activity/μg protein was assessed using paired Student’s *t* test analysis.

## Results

### TMZ treatment increases NF-κB transcriptional activity in an MMR-dependent manner

**Figure 1:**
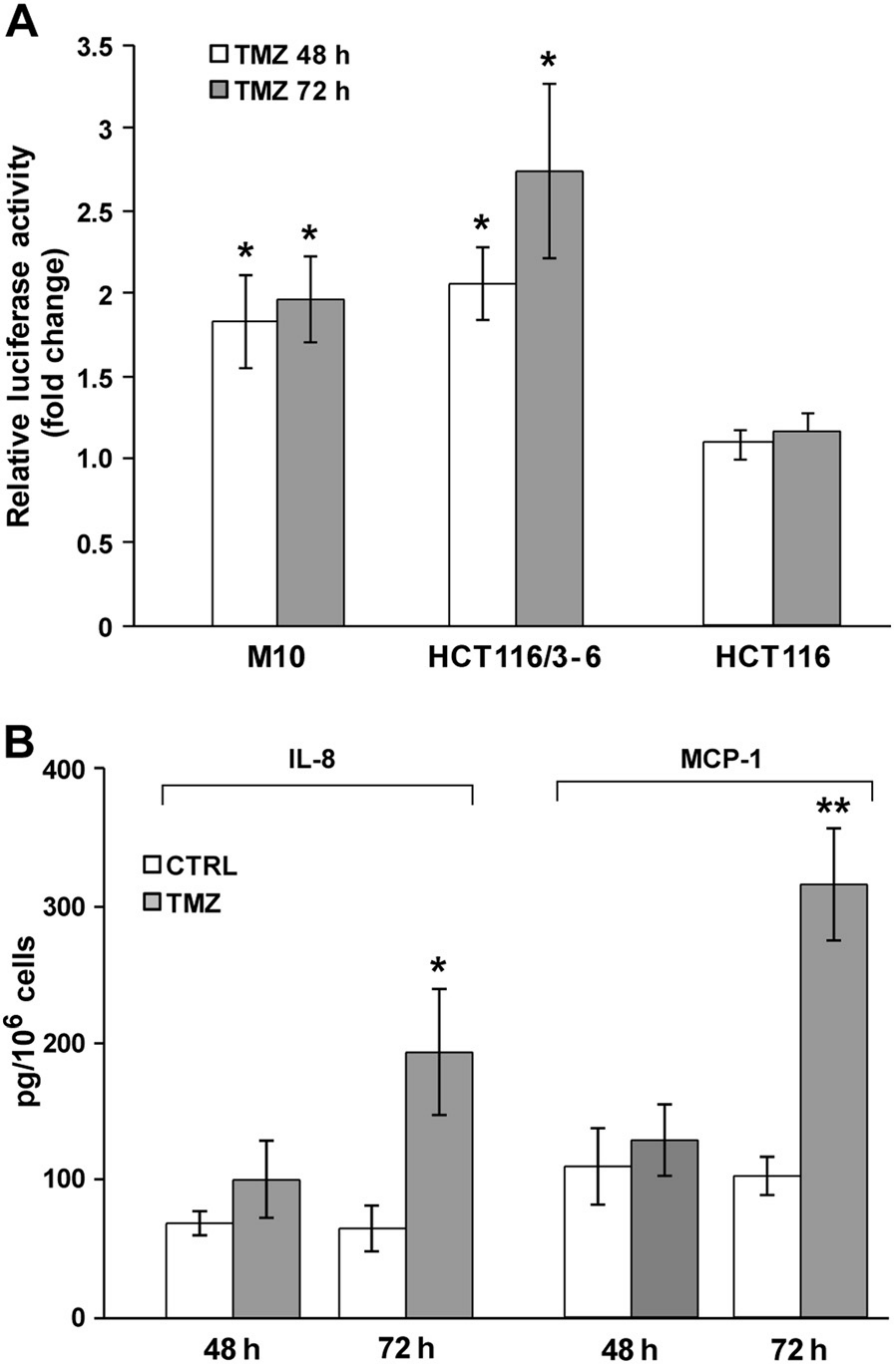
**TMZ increases NF-κB transcriptional activity in MMR-proficient but not in MMR-deficient cells.** (**A**) The cells were transiently co-transfected with the pNF-κB-Luc and pRL-null vectors, and 24 h later exposed to 50 μM TMZ+10 μM BG or to BG alone. Firefly luciferase activity was determined 48 and 72 h after drug treatment and normalized to that of *Renilla* luciferase and then to the total amount of protein used in the assays. Data are expressed in terms of fold change (i.e. the ratio between firefly luciferase activity/μg protein detected in TMZ-treated cells and that detected in the corresponding control cells). Values represent the mean of at least four independent experiments performed with duplicate samples. Bars, standard error of the mean (SEM). *p<0.05, according to paired Student’s *t* test analysis performed comparing firefly luciferase activity/μg protein of TMZ-treated samples with that of the corresponding controls. (**B**) M10 cells were cultured in the presence of 50 μM TMZ+10 μM BG or BG alone and culture supernatants were collected after 48 and 72 h of incubation. IL-8 and MCP-1 amount in the culture supernatants was then determined by ELISA and expressed in terms of pg protein/10^6^ cells. Values represent the mean of three independent experiments performed with duplicate samples. Bars, SEM. ** *p<0.01* and *p<0.05, according to Student’s *t* test analysis performed comparing the chemokine amount detected in the culture supernatants of TMZ-treated cells with that detected in the culture supernatants of the corresponding controls.

To investigate whether NF-κB transcriptional activity increases in response to TMZ and whether a functional MMR is required for this molecular event, M10, HCT116/3-6 and HCT116 cells were transfected with an NFκB-responsive luciferase reporter (pNF-κB-Luc) and then cultured in the presence of the drug. Luciferase assays performed after 48 and 72 h of exposure to TMZ evidenced a significant increase of pNF-κB-Luc reporter activity in the MMR-proficient cell lines M10 and HCT116/3-6 (Figure 1A). In contrast, no TMZ-induced change in pNF-κB-Luc reporter activity was detected in MMR-deficient HCT116 cells (Figure 1A).

*IL8* and *CCL2* genes, encoding IL-8 and MCP-1, respectively, are transcriptional targets of NF-κB [37,38]. Therefore, to further confirm the increase of NF-κB transcriptional activity in cells exposed to TMZ, we evaluated the effect of the drug on the secretion of these cytokines by M10 cells. With respect to the supernatants derived from control cultures, an increase of about 3-fold of both IL-8 and MCP-1 levels was detected in the supernatants obtained from the cultures exposed to TMZ for 72 h (Figure 1B).

### TMZ treatment induces AKT phosphorylation, IκB-α degradation and nuclear translocation of RelA in an MMR-dependent manner

**Figure 2:**
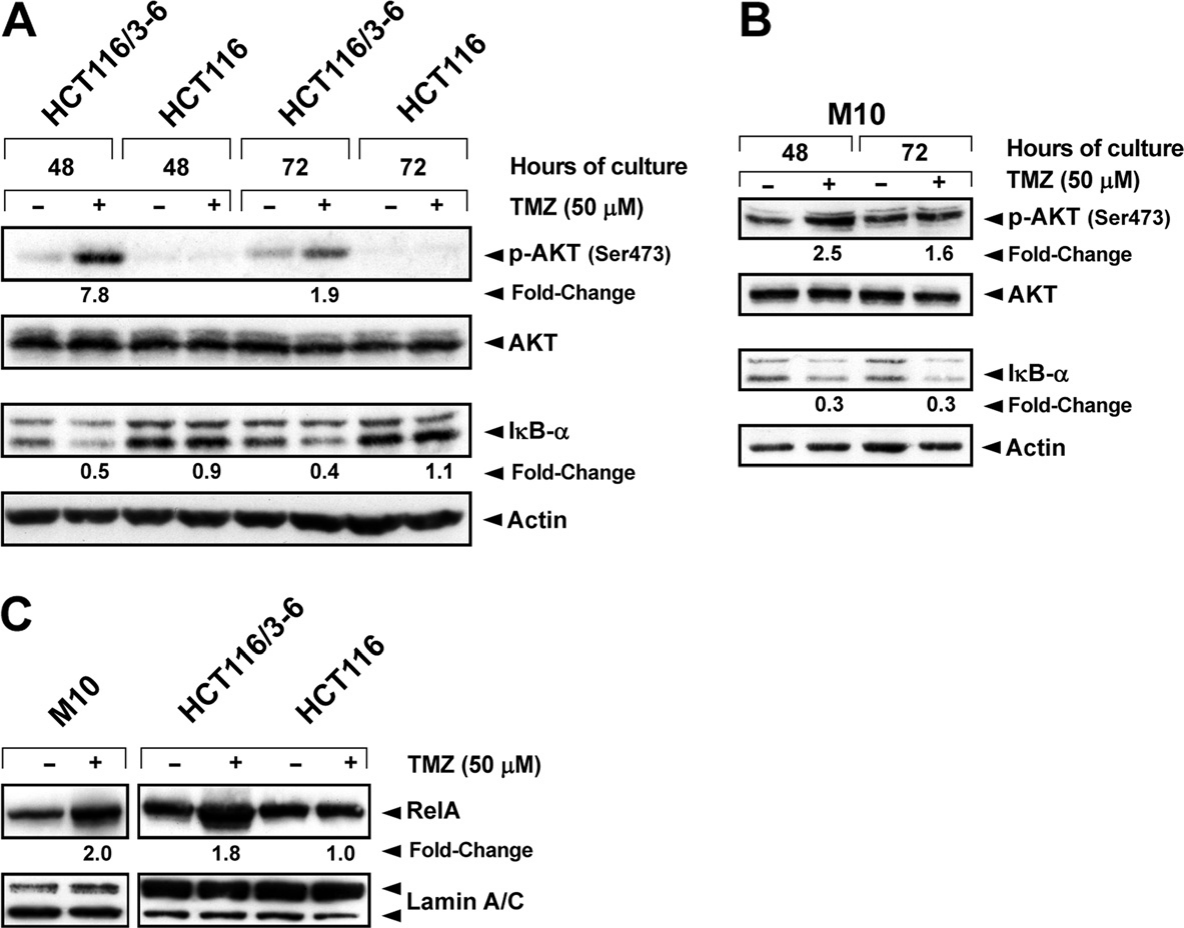
**TMZ induces AKT phosphorylation, IκB-α degradation, and RelA nuclear translocation in MMR-proficient but not in MMR-deficient cells.** HCT116/3-6, HCT116 and M10 cells were cultured in the presence of 50 μM TMZ+10 μM BG, or BG alone as a control, for 48 and 72 h (**A**, **B**) or 72 h (**C**). Total cell extracts (**A**, **B**) or nuclear extracts (**C**) were resolved on 10% SDS polyacrylamide gels, transferred to nitrocellulose membranes and probed with antibodies against the indicated proteins. Anti-actin mAb or anti-lamin A/C mAb were used for equal loading control. The immune complexes were visualized using ECL. For the indicated samples, the densitometric levels of phospho-AKT, IκB-α and RelA were normalized to the respective levels of actin or lamin. Fold changes of protein expression in TMZ-treated samples were then calculated with respect to the protein levels in the corresponding controls, to which the arbitrary value of 1.0 was assigned. The results are representative of three (**A**, **B**) or two (**C**) independent experiments.

We have previously demonstrated that in response to TMZ, AKT is phosphorylated on Ser473 and functionally activated in the MMR-proficient cell line HCT116/3-6, but not in its MMR-deficient counterpart HCT116 [25]. On the other hand, it has been shown that activation of AKT in response to several stimuli indirectly promotes IκB-α degradation and nuclear translocation of p50/RelA dimers [39-44]. On these bases, M10, HCT116/3-6 and HCT116 cells were treated with TMZ and the levels of AKT, phospho-AKT (Ser473) and IκB-α were determined after 48 and 72 h of drug exposure. In agreement with our previous data [25], TMZ-induced phosphorylation of AKT was observed in HCT116/3-6 but not in the MMR-deficient HCT116 cells (Figure 2A). An increase of phosphorylated AKT was also detected in TMZ-treated M10 cells (Figure 2B). In both HCT116/3-6 (Figure 2A) and M10 (Figure 2B) cells, but not in HCT116 cells (Figure 2A), TMZ treatment caused a reduction in the levels of IκB-α at the time points analyzed.

To examine whether nuclear translocation of NF-κB occurred in response to TMZ, RelA levels were evaluated in nuclear extracts of M10, HCT116/3-6 and HCT116 cells exposed to the drug for 72 h. In both the MMR-proficient cell lines, but not in HCT116 cells, TMZ treatment induced an increase of about two-fold of RelA nuclear content (Figure 2C).

To further confirm that TMZ-induced NF-κB activation requires a functional MMR system, nuclear translocation of RelA upon drug treatment was evaluated in an additional pair of isogenic MMR-proficient and MMR-deficient cell lines. As illustrated in Additional file 2: Figure S2, TMZ treatment increased the levels of nuclear RelA in the 293T±Lα cells cultured in the absence of doxycycline (i.e. 293TLα^+^ cells), but not in the same cells cultured in the presence of doxycycline able to turn off the expression of hMLH1 (i.e. 293TLα^-^ cells).

### TMZ treatment promotes NF-κB2/p52 generation and nuclear translocation in M10 but not in HCT116/3-6 cells

**Figure 3:**
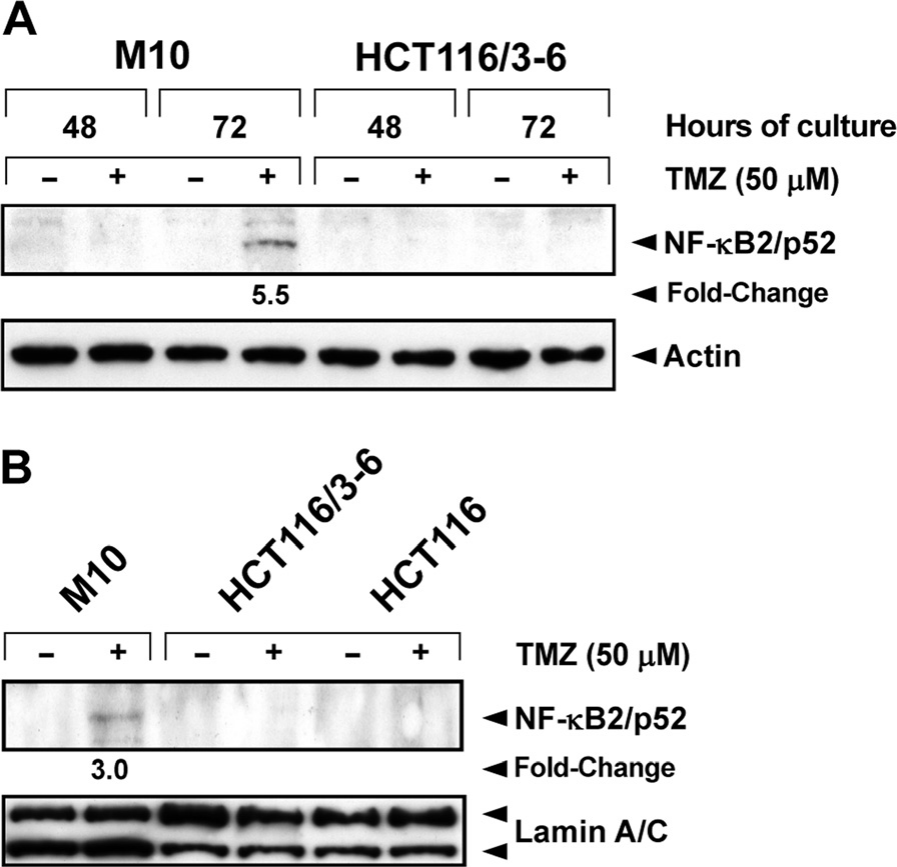
**TMZ promotes NF-κB2/p52 generation and nuclear translocation in M10 cells.** (**A**) The cells were cultured in the presence of 50 μM TMZ+10 μM BG, or BG alone for the indicated time. Total cell extracts were then prepared and analyzed for NF-κB2/p52 expression by immunoblotting (see legend of Figure 2). The results are representative of two independent experiments. (**B**) The cells were cultured as described in (**A**) for 72 h. Nuclear extracts were then prepared and analyzed for NF-κB2/p52 content as described in (**A**) using anti-lamin A/C mAb for equal loading control. The results are representative of two independent experiments.

Previous studies have demonstrated that AKT can bind and activate IKK-α leading to the processing of NF-κB2/p100 and generation of the mature NF-κB/p52 subunit [24]. We therefore investigated whether TMZ treatment was also able to stimulate NF-κB2/p52 generation and nuclear translocation in HCT116/3-6 and M10 cells. As expected, NF-κB2/p52 expression was barely detectable in total (Figure 3A) and nuclear (Figure 3B) extracts of untreated M10 and HCT116/3-6 cells. Exposure to TMZ caused an increase of total and nuclear content of NF-κB2/p52 in M10 cells, whereas it did not affect the expression or localization of the protein in HCT116/3-6 cells (Figure 3A and B).

### AKT is necessary for NF-κB activation in response to TMZ

The analysis of AKT and NF-κB activation in MMR-proficient and MMR-deficient cell lines exposed to TMZ strongly indicated that AKT could be involved in drug-induced activation of NF-κB. Additional experiments were, therefore, performed to investigate whether inhibition of AKT expression by RNA interference technology was associated with an impairment of TMZ-induced degradation of IκB-α and/or drug-induced NF-κB2/p52 generation.

**Figure 4:**
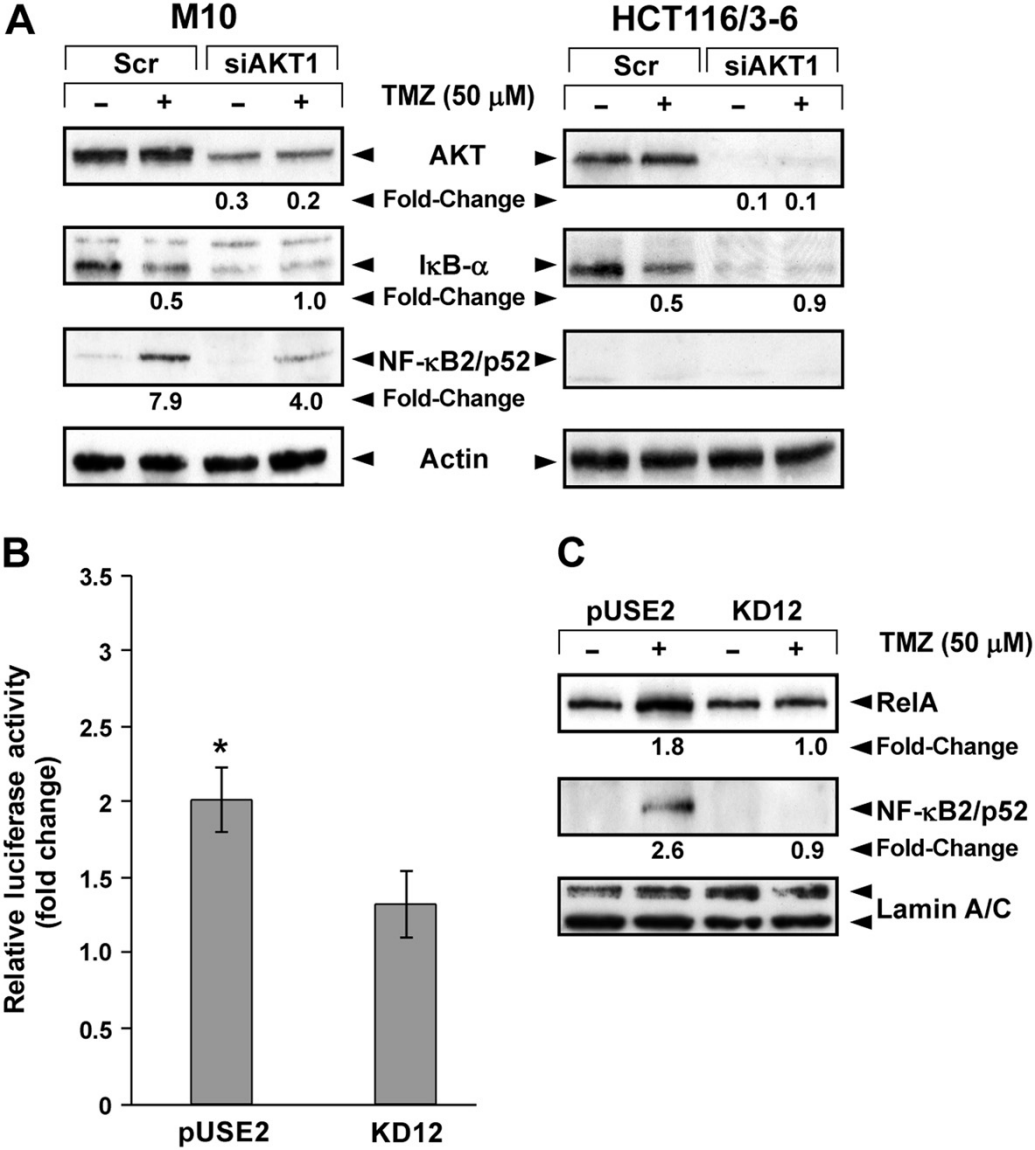
**AKT is involved in TMZ-induced activation of NF-κB.** (**A**) The cells were transiently transfected with 50 nM siAKT1 or scrAKT (Scr) and 24 h later exposed to 50 μM TMZ+10 μM BG, or to BG alone. After 72 h of culture, total cell extracts were analyzed for AKT, IκB-α and NF-κB2/p52 expression by immunoblotting (see legend of Figure 2). The results are representative of two independent experiments. (**B**) The cells were transiently co-transfected with the pNF-κB-Luc and pRL-null vectors and 24 h later incubated with 50 μM TMZ+10 μM BG or with BG alone. Luciferase assays were performed after 72 h of drug exposure. Data are expressed in terms of fold change (see legend of Figure 1). Values represent the mean of four independent experiments performed with duplicate samples. Bars, SEM. *p<0.05 according to paired Student’s *t* test analysis performed comparing firefly luciferase activity/μg protein detected in TMZ-treated pUSE2 cells with that detected in the corresponding control cells. The difference between firefly luciferase activity/μg protein of TMZ-treated KD12 cells and that detected in the corresponding control cells was not statistically significant. (**C**) The cells were treated with 50 μM TMZ+10 μM BG or with BG alone for 72 h. Nuclear extracts were then examined for the expression of RelA and NF-κB2/p52 by immunoblotting (see legend of Figure 2). The results are representative of two independent experiments.

As illustrated in Figure 4A, the amount of AKT protein was markedly reduced in siAKT1-transfected M10 and HCT116/3-6 cells as compared with their corresponding scrAKT1-transfected controls. In both M10 and HCT116/3-6 cells, AKT1 silencing suppressed IκB-α degradation in response to TMZ. Moreover, in M10 cells it also impaired drug-induced generation of NF-κB2/p52. Notably, the basal levels of IκB-α appeared strongly reduced in siAKT1-transfected cells. Since transcription of the *NFKBIA* gene, encoding IκB-α, is regulated by NF-κB [45], it is possible to hypothesize that AKT1 silencing results in a decrease of basal NF-κB activity leading to reduced transcription of the *NFKBIA* gene.

To confirm the involvement of AKT in NF-κB activation promoted by TMZ, drug-induced changes in pNF-κB-Luc reporter activity and nuclear levels of RelA and NF-kB2/p52 were evaluated in pUSE2 and KD12 cells. The former cell line expresses wild type AKT and the latter expresses a dominant-negative kinase-dead form of AKT1. Luciferase assays performed after 72 h of exposure to TMZ showed a significant increase of pNF-κB-Luc reporter activity only in pUSE2 cells (Figure 4B). Moreover, upon TMZ treatment, RelA and NF-κB2/p52 nuclear translocation was observed in pUSE2 but not in KD12 cells (Figure 4C).

### Inhibition of NF-κB increases tumor cell sensitivity to TMZ

Having demonstrated that NF-κB is activated in an AKT-dependent manner in response to TMZ, experiments were conducted to explore whether inhibition of NF-κB activity could increase TMZ sensitivity of M10 and HCT116/3-6 cells. Indeed, we have previously shown that KD12 cells, which do not activate NF-kB in response to TMZ, are approximately six-fold more sensitive to the drug than pUSE2 cells [25]. M10 and HCT116/3-6 cells were mock-transfected or transfected with a siRNA targeting RelA (siNFp65) or a scrambled siRNA (scrNFp65) and their sensitivity to TMZ was evaluated by the MTT assay.

**Figure 5:**
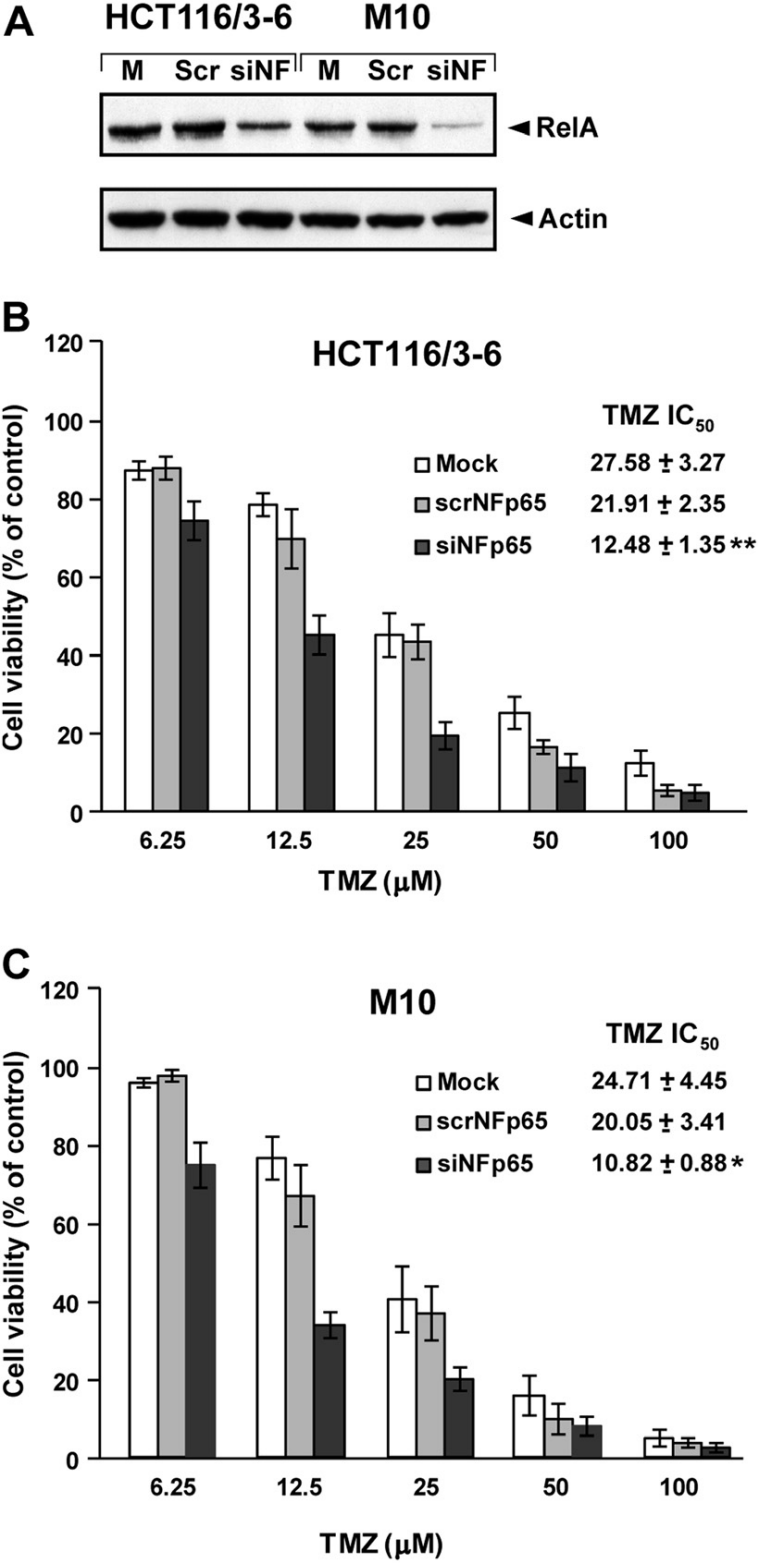
**Inhibition of NF-κB expression by RNA interference increases HCT116/3-6 and M10 cell sensitivity to TMZ.** (**A**) The cells were mock transfected (M) or subjected to two sequential transfections with 100 nM scrNFp65 (Scr) or siNFp65 (siNF). Seven days after the second transfection, whole cell extracts were prepared and analyzed for RelA content by immunoblotting (see legend of Figure 2). The results are representative of two independent experiments. (**B**), (**C**) The cells were mock transfected or subjected to two sequential transfections with 100 nM scrNFp65 or siNFp65. Twenty-four hours after the second transfection, the cells were incubated with the indicated concentrations of TMZ+10 μM BG or with BG alone as a control. Proliferation was evaluated after 6 days of drug exposure by the MTT assay. Data are expressed in terms of percentage of growth of TMZ-treated cells with respect to control cells. Each value represents the mean of four independent experiments performed with quadruplicate samples. Bars, SEM. **p<0.01, and *p<0.05, according to Student’s *t* test, comparing TMZ IC_50_ values of siNFp65-transfected cells with those of mock-transfected or scrNFp65-transfected cells. The difference between TMZ IC_50_ values of scrNFp65 and those of mock-transfected cells was not statistically significant.

With respect to mock-transfected cells, a clear reduction of RelA levels was observed in siNFp65-transfected cells, whereas the protein amount was unchanged in scrNFp65-transfected cells (Figure 5A). Moreover, inhibition of RelA expression was accompanied by a significant enhancement of HCT116/3-6 and M10 cell sensitivity to TMZ (Figures 5B and C).

The NBD peptide is a cell permeable peptide spanning the NBD of IKK-β and able to disrupt the NEMO-IKK complex interaction [35]. In a first set of experiments, we therefore investigated whether the NBD peptide was able to impair TMZ-induced activation of NF-κB in M10 cells and to increase their sensitivity to the drug.

**Figure 6:**
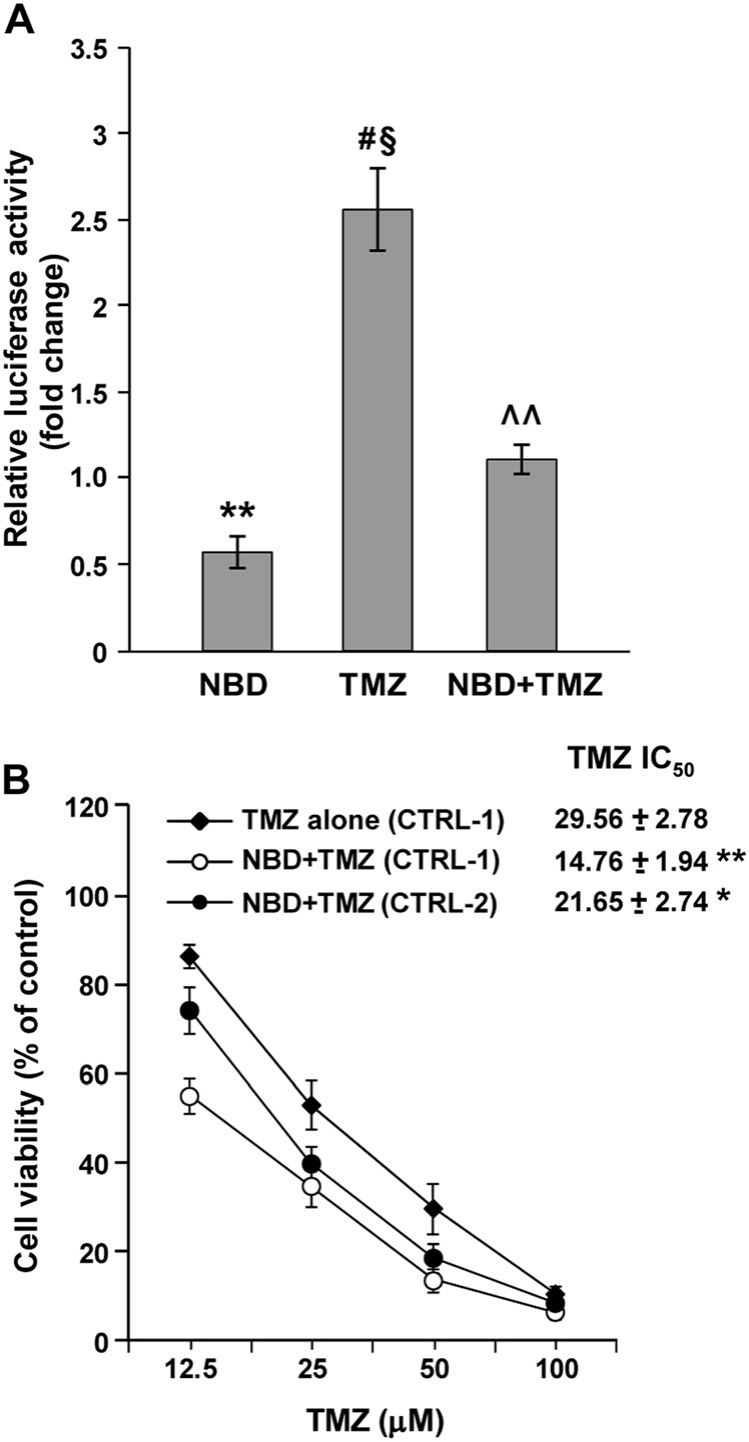
**NBD peptide inhibits NF-κB transcriptional activity and increases the antiproliferative effect of TMZ in M10 cells.** (**A**) Melanoma cells were transiently co-transfected with the NF-κB-Luc and pRL-null vectors and treated with NBD peptide, TMZ and BG as described under *“Methods”.* Luciferase assays were performed after 72 of drug exposure. Data are expressed in terms of fold changes (i.e. the ratio between firefly luciferase activity/μg protein detected in cells exposed to NBD+BG, or TMZ+BG or NBD+TMZ+BG and that detected in cells treated with BG alone). Values represent the mean of six independent experiments performed with duplicate samples. Bars, SEM. Statistical analysis was performed on firefly luciferase activity/μg protein values according to paired Student’s *t* test. *p* values were as follows: **p<0.01, NBD+BG versus BG; ^#^*p<0.05*, TMZ+BG versus BG; ^§^*p<0.05* TMZ+BG versus NBD+TMZ+BG; ^^*p<0.01*, NBD+TMZ+BG versus NBD+BG. The differences between firefly luciferase activity/μg protein of cells treated with NBD+TMZ+BG and that of cells exposed to BG alone was not statistically significant. (**B**) M10 cells were treated with NBD peptide, TMZ and BG as described under *“Methods”* and assayed for proliferation by the MTT assay. Percentage of growth of TMZ+BG-treated cells was determined with respect to BG-treated cells (CTRL-1), and that of NBD+TMZ+BG-treated cells with respect to either BG-treated cells (CTRL-1) or NBD+BG-treated cells (CTRL-2). Values represent the mean of four independent experiments. Bars, SEM. **p<0.01, and *p<0.05, according to Student’s *t* test analysis, comparing TMZ IC_50_ values of NBD+TMZ+BG-treated cells with those of TMZ+BG-treated cells.

Luciferase assays showed that the pNF-kB-Luc reporter activity detected in M10 cells exposed to NBD peptide for 96 h was significantly lower than that observed in control cells (Figure 6A). Moreover, NBD peptide was able to suppress TMZ-induced activation of NF-κB (Figure 6A). As illustrated in Figure 6B, a significant increase in the growth suppressive effects of TMZ was observed when the drug was used in association with the NBD peptide. Indeed, the IC_50_ values of TMZ displayed by the cells exposed to NBD peptide were significantly lower than those displayed by the cells not treated with the peptide. This was true not only when the IC_50_ values were determined with respect to control cells treated with BG alone (CTRL-1), but also when they were evaluated with respect to control cells exposed to NBD peptide+BG (CTRL-2).

Previous studies have demonstrated that TMZ induces senescence but not apoptosis in human melanoma cells [46]. We therefore investigated whether the combined treatment with NBD peptide and TMZ was more effective than TMZ alone in inducing senescence in M10 cells.

**Figure 7:**
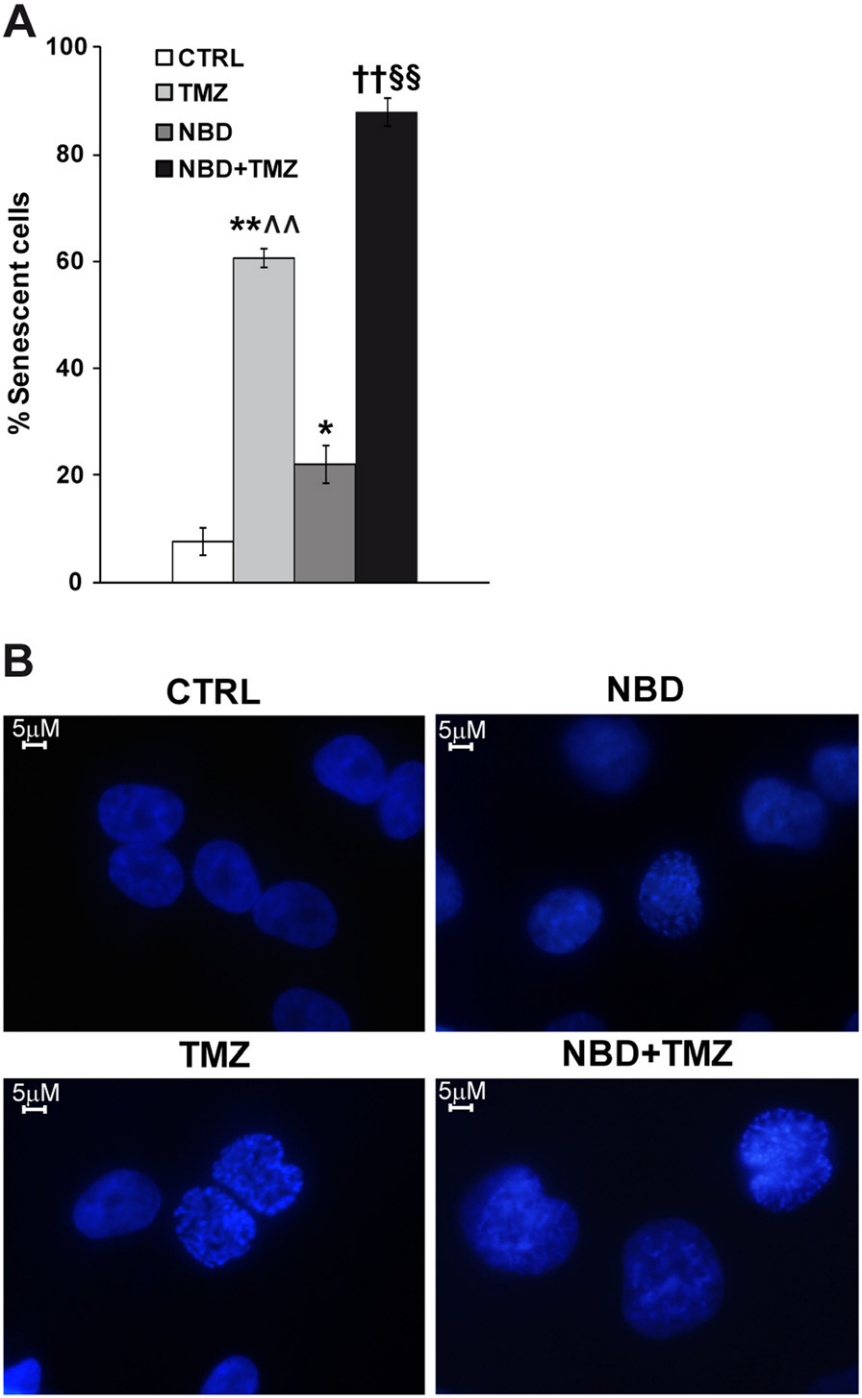
**Combined treatment with NBD peptide and TMZ is more effective than TMZ alone in inducing senescence in M10 cells.** (**A**) M10 cells were left untreated or exposed to 50 μM NBD peptide. After 24 h of culture, the cells were incubated with 50 μM TMZ+10 μM BG or with BG alone, and monitored 96 h later for the percentage of SA-β-Gal positive cells. Each value represents the arithmetic mean of three independent experiments. Bar, SEM. *p* values were calculated according to Student’s *t* test analysis. **p<0.01, TMZ+BG-treated cells versus BG-treated cells; *p<0.05, NBD peptide+BG-treated cells versus BG-treated cells; ^††^*p<0.01*, NBD peptide+TMZ+BG-treated cells versus BG-treated cells; ^§§^*p<0.01* NBD peptide+TMZ+BG-treated cells versus TMZ+BG-treated cells and versus NBD+BG-treated cells; ^^*p<0.01*, TMZ+BG-treated cells versus NBD peptide+BG cells. (**B**) M10 cells were treated with NBD peptide and TMZ+BG as described in (A). After 7 days of culture, the cells were stained with DAPI to visualize SAHF formation.

The results illustrated in Figure 7A show that treatment with NBD peptide caused a moderate, although significant, increase in the percentage of SA-β-Gal positive cells whereas about 60% of the cells appeared senescent in TMZ-treated cultures. Consistent with the results of proliferation assays, the proportion of SA-β-Gal positive cells detected in the cultures subjected to the combined treatment with NBD peptide and TMZ was significantly higher than that displayed by the cultures exposed to each drug separately. Induction of senescence in M10 cells treated with TMZ or NBD peptide or the combination of both agents, was also confirmed by the appearance of SAHF in these cells. As shown in Figure 7B, control cells displayed an uniform pattern of DAPI staining, whereas drug-treated cells exhibited punctuated DAPI foci, indicative of SAHF formation.

The growth suppressive effects of the association of NBD peptide and TMZ were also evaluated in the HCT116/3-6 cell line. Even in this case the association of NBD peptide and TMZ was more effective than TMZ alone in suppressing cell growth (Additional file 3: Figure S3A) and inducing senescence (Additional file 3: Figure S3B and S3C). However, in HCT116/3-6 cells, the effects of the two drugs appear to be additive.

## Discussion

Constitutive activation of NF-κB is frequently observed in different types of cancer and has been correlated with tumor development, progression and radio- and chemoresistance. Actually, NF-κB regulates the expression of a wide array of genes that actively participate in controlling cell proliferation and survival, as well as angiogenesis and metastasis [1-3]. Targeting either activation or function of NF-κB is therefore considered a promising strategy to inhibit tumor growth and metastasis and to increase the efficiency of therapy [1-3].

A growing body of experimental evidence indicates that, in addition to the large number of signals that act through membrane and/or cytoplasmic receptors, various DNA-damaging chemotherapeutic agents can lead to NF-κB activation through initiating signals generated in the nucleus [5-7]. However, the final outcome of drug-induced up-regulation of NF-κB pathway, i.e. cell protection from or sensitization to the growth suppressive and/or death promoting effects of the drug, appears to be dependent on the cell type and/or the nature and the amount of the agent [5-7,47-50]. The dual role of NF-κB activation in the modulation of tumor cell response to chemotherapeutic agents appears to result from the ability of this transcription factor to either activate or repress transcription of genes involved in cell proliferation and survival, depending on which specific post-translational modifications and interaction with transcription co-activator or repressors occur in the malignant cells [1,3,23,51]. Therefore, the final outcome of strategies targeting NF-κB to increase cancer cell response to chemotherapy should be careful validated for each tumor type and anticancer agent.

In this study, we demonstrate that exposure of MMR-proficient malignant cells to a clinical relevant concentration of TMZ caused activation of the NF-κB signalling pathway. Indeed, using a NF-κB-responsive luciferase reporter, a 2-3-fold increase of NF-κB transactivation function was detected in HCT116/3-6, pUSE2 and M10 cells treated with the drug. Consistent with this finding, exposure to TMZ was able to enhance the secretion of IL-8 and MCP-1, two NF-κB regulated cytokines, in M10 cells. Notably, the expression of both IL-8 and MCP-1 has been associated with melanoma progression by affecting the growth of tumor cells, angiogenesis and metastasis [52].

In both HCT116/3-6, and M10 cells, degradation of IκB-α and nuclear translocation of RelA were detected after exposure to TMZ. An increase in the nuclear content of RelA was also observed in drug-treated 293TLα^+^ cells. Moreover, drug treatment stimulated the generation and nuclear accumulation of the NF-κB2/p52 subunit in M10 cells. These findings indicate that TMZ-induced activation of NF-κB can occur via the non-canonical and/or the canonical pathway, depending on the cell line. At present, the cellular characteristics that allow NF-κB activation via the non-canonical pathway upon cell exposure to TMZ remain to be established. Previous studies have shown that the expression of IKK-α and IKK-β as well as the proportion of their homo- and heterodimers vary among different cell types [53]. It is possible to speculate that the engagement of the non-canonical pathway of NF-κB activation by TMZ depends, at least in part, on the levels of IKK-α homodimer present in the cells.

TMZ-induced activation of NF-κB appears to be strictly dependent on a functional MMR system. Moreover, AKT activation downstream the MMR system appears to be involved in TMZ-induced triggering of both the canonical and non-canonical pathway of NF-κB activation. Indeed, the MMR-deficient cell line HCT116 failed to activate AKT and to degrade IκB-α in response to the drug. Accordingly, HCT116 cells did not show any increase of pNF-κB-Luc reporter activity and RelA nuclear levels upon TMZ treatment. Furthermore, in the MMR-proficient cell lines HCT116/3-6 and M10, which activate AKT in response to TMZ, impairment of AKT1 expression by RNA interference markedly inhibited drug-induced degradation of IκB-α and, in the case of M10 cells, also drug-induced enhancement of NF-κB2/p52 levels. Finally, TMZ was able to increase NF-κB-dependent luciferase activity and to induce nuclear translocation of RelA and NF-κB2/p52 in the MMR-proficient cell line pUSE2 but not in the isogenic KD12 cells, which express a dominant-negative kinase-dead form of AKT1.

Previous investigations have demonstrated that AKT, via different down-stream effector molecules, can promote IκB-α degradation and p50/RelA or p50/RelB nuclear translocation and can enhance the transactivation potential of RelA [43,44,54,55]. Moreover, it has been shown that AKT can directly phosphorylate IKK-α leading to the processing of p100 and nuclear accumulation of p52/RelB dimers [24]. Our data are consistent with these findings, even though further studies are required to detail further the molecular mechanisms underlying AKT-dependent NF-κB activation in TMZ-treated cells.

Regardless of the mechanisms involved in AKT-dependent activation of NF-κB in TMZ-treated cells, our results show that this molecular event has a pro-survival function in tumor cells presenting constitutive activation of the MAPK and/or PI3K/AKT signaling pathways. Indeed, KD12 cells, which do not activate NF-κB in response to TMZ are significantly more sensitive to the drug than pUSE2 cells [25]. Moreover, impairment of RelA expression by RNA interference enhanced HCT116/3-6 and M10 cell sensitivity to TMZ. Similarly, an increase of TMZ growth suppressive effect was observed in M10 cells when the drug was associated with a concentration of NBD peptide able to attenuate basal NF-κB activity and to abrogate TMZ-induced up-regulation of NF-κB activity. In these cells, combined treatment with NBD peptide and TMZ induced higher levels of senescence as compared with those caused by each drug alone. Notably, in agreement with previous studies [56] and with the role of NF-κB signaling pathway in melanoma growth and survival [57,58], we found that NBD peptide, as a single agent, was able to impair melanoma cell proliferation in a concentration-dependent manner. However, it must be pointed out that the NBD peptide concentration exploited in combination with TMZ only moderately affected M10 cell proliferation when used alone. Moreover, TMZ IC_50_ values determined in presence of the NBD peptide were significantly lower than those determined in the absence of the NF-κB inhibitor, not only when they were evaluated using BG-treated cells as control group, but also when they were calculated using BG+NBD-treated cells as control group. These findings show that TMZ is more efficient in M10 cells when associated with the NF-κB inhibitor. Notably, the same drug combination was also found to be more active than TMZ alone in HCT116/3-6 cells, although NBD peptide + TMZ showed only additive effects on cell growth.

So far, a small number of studies have addressed the effects of TMZ on NF-κB pathway with some discordant results. Amiri et al. [59] demonstrated that exposure to TMZ increased RelA nuclear localization in melanoma cells and that bortezomib, an inhibitor of proteasome able to impair NF-κB activation [60], potentiated TMZ-induced suppression of melanoma cell growth both *in vitro* and in a murine xenograft model. Using an NF-κB-responsive luciferase reporter, Ohanna et al. [61] also observed NF-κB activation in melanoma cells exposed to TMZ. On the other hand, Yamini et al. [62] and Schmitt et al. [63] demonstrated that TMZ treatment impaired NF-κB transcriptional activity in glioblastoma cells. In these cells, TMZ-induced activation of Chk1 led to phophorylation of the NF-κB1/p50 subunit on Ser329, disrupting the DNA binding ability of NF-κB dimers containing this subunit [63].

The results of our study are consistent with the findings of Amiri et al. [59] and Ohanna et al. [61]. However, we also show for the first time that, depending on the cell line, both the canonical and non-canonical pathway of NF-κB activation can be elicited by TMZ and that AKT plays a crucial role in the drug-induced activation of both pathways. On the other hand, the discrepancy between our results and those of Yamini et al. [62] and Schmitt et al. [63] might depend on the different cellular models utilized to assess the effect of TMZ on NF-κB activity. Interestingly, Hirose et al. [64] have shown that in glioblastoma cells TMZ treatment causes a decrease in the endogenous levels of AKT phosphorylated on Ser473. Previous investigations have also shown that activated AKT is able to phosphorylate Chk1 on Ser280, leading to inactivation and cytoplasmic localization of this checkpoint kinase [65-67]. It is possible to speculate that TMZ treatment could result in NF-κB activation or inhibition, depending on whether in the cell line under investigation the drug activates or not AKT in addition to Chk1. However, further studies are required to address this issue.

## Conclusions

The present investigation demonstrates that in MMR-proficient tumor cell lines of various histological derivation, NF-κB is activated in response to clinical relevant concentrations of TMZ. Our study also provides, for the first time, experimental evidence that AKT is involved in TMZ-induced activation of NF-κB and that this molecular event can occur through both the canonical and non-canonical pathway. Finally, our results support a pro-survival role of TMZ-induced activation of NF-κB. Several specific inhibitors of the NF-κB pathway are currently being developed and will likely enter clinical trials in the near future, either as single agents or in combination with conventional chemotherapy [1-3,58]. Our findings strongly suggest that a clinical benefit could be obtained by combining TMZ with this type of biochemical modulators.

## Abbreviations

ATM: Ataxia-Telangiectasia Mutated; BG: O^6^-benzylguanine; Chk1: Checkpoint Kinase 1; CTRL: Control; DAPI: 4’-6-Diamidino-2-phenylindol; ECL: Enhanced chemiluminescence; ELISA: Enzyme-linked immunosorbent assay; FCS: Fetal calf serum; IC_50_: Concentration producing 50% inhibition; IκBs: Inhibitors of κB; IKK: IκB kinase; IL-8: Interleukin 8; mAb: Monoclonal antibody; MAPK: Mitogen activated protein kinase; MCP-1: Monocyte chemoattractant protein-1; MGMT: O^6^-methylguanine-DNA methyltransferase; MMR: Mismatch repair; MTT: 3-(4,5-dimethylthiazol-2-yl)-2,5-diphenyltetrazolium bromide; NBD: NEMO-binding domain; NEMO: NF-κB essential modulator; NF-κB: Nuclear factor κB; O^6^-MeG: O^6^-methylguanine; PBS: Phosphate buffered saline; PI3K: Phosphatidylinositol-3-kinase; SA-β-Gal: Senescence associated β-galactosidase; SAHF: Senescence-associated heterochromatin foci; SDS: Sodium dodecyl sulfate; SEM: Standard error of the mean; siRNA: Small interfering RNA; TMZ: Temozolomide; TNF: Tumor necrosis factor.

## Competing interests

Dr Paolo A. Ascierto stood on the Advisory Board of Bristol Myers Squibb, Merck Sharp & Dohme, Roche-Genentech, GSK and Amgen, and received honoraria from Bristol Myers Squibb, Merck Sharp & Dohme, and Roche-Genentech. All remaining authors declare that they have no competing interests.

## Authors’ contributions

SC was involved in the study conception, design of the experiments, acquisition, analysis and interpretation of the data concerning TMZ-induced modulation of NF-κB transcriptional activity, and the effects of inhibiting NF-κB activity on cell sensitivity to the drug. She drafted the manuscript. LL and MGA carried out the transient transfections with siRNAs and the Western blot analyses. GG and AM were involved in the acquisition, analysis and interpretation of the data concerning the effect of TMZ on IL-8 and MCP-1 secretion. GG, EB, GP, and PAA contributed to the study discussion and to the revision of the manuscript. SD was involved in the study conception, design and coordination, supervised data interpretation and revised the manuscript. All authors read and approved the final manuscript.

## Supplementary Material

Additional file 1 **Figure S1.** Mechanisms of O^6^-MeG-dependent growth suppression. Click here for file

Additional file 2 **Figure S2.** TMZ induces nuclear translocation of RelA in an MMR-dependent manner in 293T±Lα cells. 293T±Lα cells were cultured in the absence (293TLα^+^ cells) or in the presence (293TLα^-^ cells) of 50 ng/ml doxycyclin for 7 days and then exposed to 2 μM TMZ for 72 h. The TMZ concentration used was selected on the basis of the high susceptibility of these cells to TMZ (IC_50_: 1.27±0.14 μM, as obtained in MTT assays). Nuclear extracts were prepared, resolved on 10% SDS polyacrylamide gels, transferred to nitrocellulose membranes and probed with antibodies against RelA. Anti-lamin A/C mAb was used for equal loading control. The immune complexes were visualized using ECL. Fold changes of RelA expression in TMZ-treated samples were calculated as described in the legend of Figure 2. The results are representative of two independent experiments. Click here for file

Additional file 3 **Figure S3.** Effect of the NBD peptide on TMZ sensitivity of HCT116/3-6 cells. (**A**) The cells were left untreated or incubated with 50 μM NBD peptide for 24 h and then exposed to increasing concentrations of TMZ plus 10 μM BG or to BG alone. After additional 5 days of culture, cell proliferation was evaluated by the MTT assay. Data are expressed in terms of percentage of growth, calculated for TMZ+BG-treated cells with respect to BG-treated cells (CTRL-1), and for NBD+TMZ+BG-treated cells with respect to either BG-treated cells (CTRL-1) or NBD+BG-treated cells (CTRL-2). Each value represents the mean of three independent experiments performed with quadruplicate samples. Bars, SEM. **p<0.01, according to Student’s *t* test analysis, comparing TMZ IC_50_ values of NBD+TMZ+BG-treated cells with those of TMZ+BG-treated cells. (**B**) The cells were left untreated or exposed to 50 μM NBD peptide. After 24 h of culture, the cells were incubated with 50 μM TMZ+10 μM BG or with BG alone and monitored 96 h later for the percentage of SA-β-Gal positive cells. Each value represents the arithmetic mean of three independent experiments. Bar, SEM. *p* values were calculated according to Student’s *t* test analysis. **p<0.01, TMZ+BG-treated cells and NBD peptide+BG-treated cells versus BG-treated cells; ^††^*p<0.01*, NBD peptide+TMZ+BG-treated cells versus BG-treated cells; ^§§^*p<0.01* NBD peptide+TMZ+BG-treated cells versus TMZ+BG-treated cells and versus NBD+BG-treated cells; ^^*p<0.01*, TMZ+BG-treated cells versus NBD peptide+BG cells. (**C**) HCT116/3-6 cells were treated with NBD peptide and TMZ+BG as described in (B). After 7 days of culture, the cells were stained with DAPI to visualize SAHF formation. Click here for file
