# Supplementary figures and images for: NF-κB is activated in response to temozolomide in an AKT-dependent manner and confers protection against the growth suppressive effect of the drug
Source: J Transl Med. 2012 Dec 21;10:252. doi: 10.1186/1479-5876-10-252 (PMC3551789; doi:10.1186/1479-5876-10-252)

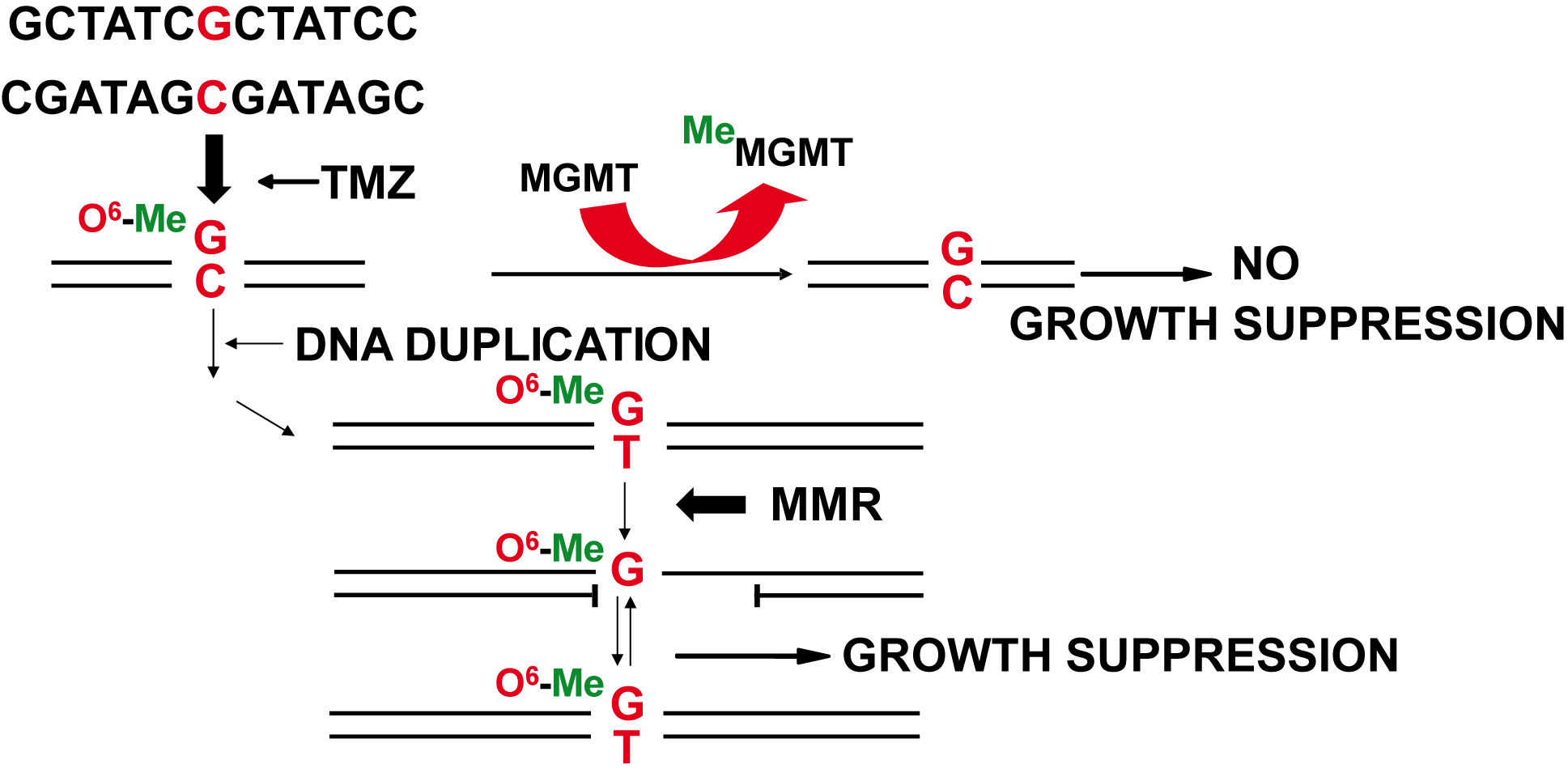

Supplement: Additional file 1 — Figure S1. Mechanisms of O6-MeG-dependent growth suppression. [file 1479-5876-10-252-S1.tiff]

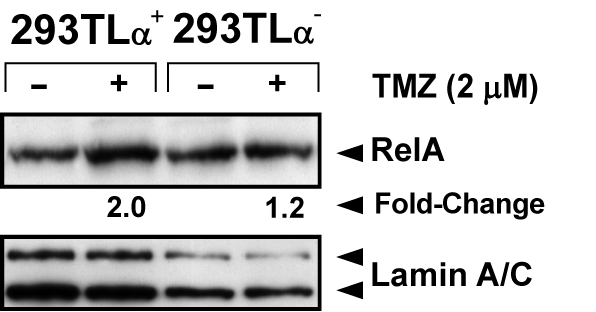

Supplement: Additional file 2 — Figure S2. TMZ induces nuclear translocation of RelA in an MMR-dependent manner in 293T±Lα cells. 293T±Lα cells were cultured in the absence (293TLα+ cells) or in the presence (293TLα- cells) of 50 ng/ml doxycyclin for 7 days and then exposed to 2 μM TMZ for 72 h. The TMZ concentration used was selected on the basis of the high susceptibility of these cells to TMZ (IC50: 1.27±0.14 μM, as obtained in MTT assays). Nuclear extracts were prepared, resolved on 10% SDS polyacrylamide gels, transferred to nitrocellulose membranes and probed with antibodies against RelA. Anti-lamin A/C mAb was used for equal loading control. The immune complexes were visualized using ECL. Fold changes of RelA expression in TMZ-treated samples were calculated as described in the legend of Figure 2. The results are representative of two independent experiments. [file 1479-5876-10-252-S2.tiff]

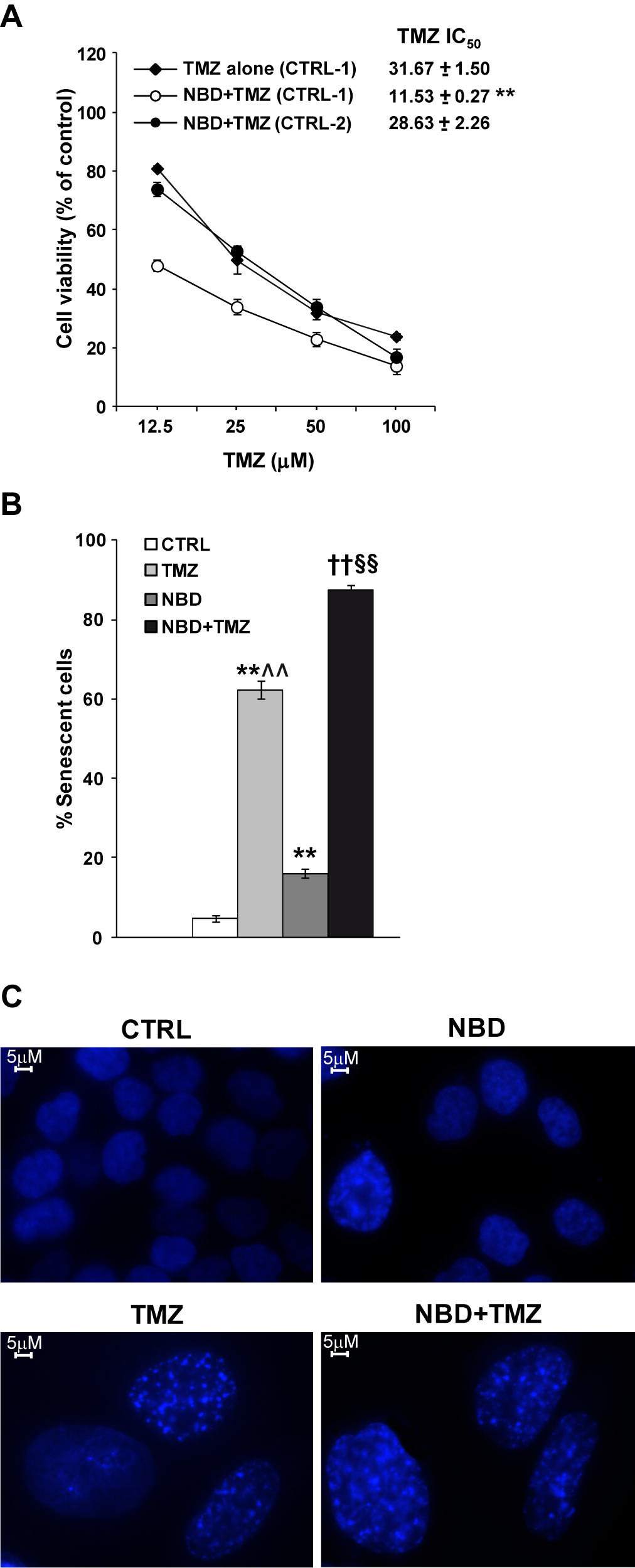

Supplement: Additional file 3 — Figure S3. Effect of the NBD peptide on TMZ sensitivity of HCT116/3-6 cells. (A) The cells were left untreated or incubated with 50 μM NBD peptide for 24 h and then exposed to increasing concentrations of TMZ plus 10 μM BG or to BG alone. After additional 5 days of culture, cell proliferation was evaluated by the MTT assay. Data are expressed in terms of percentage of growth, calculated for TMZ+BG-treated cells with respect to BG-treated cells (CTRL-1), and for NBD+TMZ+BG-treated cells with respect to either BG-treated cells (CTRL-1) or NBD+BG-treated cells (CTRL-2). Each value represents the mean of three independent experiments performed with quadruplicate samples. Bars, SEM. **p<0.01, according to Student’s t test analysis, comparing TMZ IC50 values of NBD+TMZ+BG-treated cells with those of TMZ+BG-treated cells. (B) The cells were left untreated or exposed to 50 μM NBD peptide. After 24 h of culture, the cells were incubated with 50 μM TMZ+10 μM BG or with BG alone and monitored 96 h later for the percentage of SA-β-Gal positive cells. Each value represents the arithmetic mean of three independent experiments. Bar, SEM. p values were calculated according to Student’s t test analysis. **p<0.01, TMZ+BG-treated cells and NBD peptide+BG-treated cells versus BG-treated cells; ††p<0.01, NBD peptide+TMZ+BG-treated cells versus BG-treated cells; §§p<0.01 NBD peptide+TMZ+BG-treated cells versus TMZ+BG-treated cells and versus NBD+BG-treated cells; ^^p<0.01, TMZ+BG-treated cells versus NBD peptide+BG cells. (C) HCT116/3-6 cells were treated with NBD peptide and TMZ+BG as described in (B). After 7 days of culture, the cells were stained with DAPI to visualize SAHF formation. [file 1479-5876-10-252-S3.tiff]
